# Antibody-free measurement of cerebrospinal fluid tau phosphorylation across the Alzheimer’s disease continuum

**DOI:** 10.1186/s13024-022-00586-0

**Published:** 2022-12-12

**Authors:** Johan Gobom, Andréa L. Benedet, Niklas Mattsson-Carlgren, Laia Montoliu-Gaya, Nina Schultz, Nicholas J. Ashton, Shorena Janelidze, Stijn Servaes, Mathias Sauer, Tharick A. Pascoal, Thomas K. Karikari, Juan Lantero-Rodriguez, Gunnar Brinkmalm, Henrik Zetterberg, Oskar Hansson, Pedro Rosa-Neto, Kaj Blennow

**Affiliations:** 1 Department of Psychiatry and Neurochemistry, Institute of Neuroscience and Physiology, The Sahlgrenska Academy, University of Gothenburg, Gothenburg, Sweden; 2 Clinical Neurochemistry Laboratory, Sahlgrenska University Hospital, Mölndal, Sweden; 3 Translational Neuroimaging Laboratory, McGill Centre for Studies in Aging, McGill University, Montreal, QC Canada; 4 Clinical Memory Research Unit, Faculty of Medicine, Lund University, Lund, Sweden; 5 Department of Neurology, Skåne University Hospital, Lund University, Lund, Sweden; 6 Wallenberg Centre for Molecular and Translational Medicine, University of Gothenburg, Gothenburg, Sweden; 7 King’s College London, Institute of Psychiatry, Psychology & Neuroscience, Maurice Wohl Clinical Neuroscience Institute, London, UK; 8 NIHR Biomedical Research Centre for Mental Health & Biomedical Research Unit for Dementia at South London & Maudsley NHS Foundation, London, UK; 9 Department of Psychiatry, University of Pittsburgh, Pittsburgh, PA USA; 10 Department of Neurodegenerative Disease, UCL Institute of Neurology, Queen Square, London, UK; 11 UK Dementia Research Institute at UCL, London, UK; 12 Hong Kong Center for Neurodegenerative Diseases, Hong Kong, China; 13 Memory Clinic, Skåne University Hospital, Lund University, Lund, Sweden

**Keywords:** Tau, Phosphorylation, Alzheimer’s disease, LC–MS, Cerebrospinal fluid

## Abstract

**Background:**

Alzheimer’s disease is characterized by an abnormal increase of phosphorylated tau (pTau) species in the CSF. It has been suggested that emergence of different pTau forms may parallel disease progression. Therefore, targeting multiple specific pTau forms may allow for a deeper understanding of disease evolution and underlying pathophysiology. Current immunoassays measure pTau epitopes separately and may capture phosphorylated tau fragments of different length depending on the non-pTau antibody used in the assay sandwich pair, which bias the measurement.

**Methods:**

We developed the first antibody-free mass spectrometric method to simultaneously measure multiple phosphorylated epitopes in CSF tau: pT181, pS199, pS202, pT205, pT217, pT231, and pS396. The method was first evaluated in biochemically defined Alzheimer’s disease and control CSF samples (*n* = 38). All seven pTau epitopes clearly separated Alzheimer’s disease from non-AD (*p* < 0.001, AUC = 0.84–0.98). We proceeded with clinical validation of the method in the TRIAD (*n* = 165) and BioFINDER-2 cohorts (*n* = 563), consisting of patients across the full Alzheimer’s disease *continuum*, including also young controls (< 40 years), as well as patients with frontotemporal dementia and other neurodegenerative disorders.

**Results:**

Increased levels of all phosphorylated epitopes were found in Alzheimer’s disease dementia and Aβ positron emission tomography-positive patients with mild cognitive impairment compared with Aβ-negative controls. For Alzheimer’s disease dementia compared with Aβ-negative controls, the best biomarker performance was observed for pT231 (TRIAD: AUC = 98.73%, fold change = 7.64; BioFINDER-2: AUC = 91.89%, fold change = 10.65), pT217 (TRIAD: AUC = 99.71%, fold change = 6.33; BioFINDER-2: AUC = 98.12%, fold change = 8.83) and pT205 (TRIAD: AUC = 99.07%, fold change = 5.34; BioFINDER-2: AUC = 93.51%, fold change = 3.92). These phospho-epitopes also discriminated between Aβ-positive and Aβ-negative cognitively unimpaired individuals: pT217 (TRIAD: AUC = 83.26, fold change = 2.39; BioFINDER-2: AUC = 91.05%, fold change = 3.29), pT231 (TRIAD: AUC = 86.25, fold change = 3.80; BioFINDER-2: AUC = 78.69%, fold change = 3.65) and pT205 (TRIAD: AUC = 71.58, fold change = 1.51; BioFINDER-2: AUC = 71.11%, fold change = 1.70).

**Conclusions:**

While an increase was found for all pTau species examined, the highest fold change in Alzheimer’s disease was found for pT231, pT217 and pT205. Simultaneous antibody-free measurement of pTau epitopes by mass spectrometry avoids possible bias caused by differences in antibody affinity for modified or processed forms of tau, provides insights into tau pathophysiology and may facilitate clinical trials on tau-based drug candidates.

**Supplementary Information:**

The online version contains supplementary material available at 10.1186/s13024-022-00586-0.

## Background

Abnormally phosphorylated tau (pTau) has been a focus of Alzheimer’s disease research since the discovery of these species as the main constituent of the intraneuronal neurofibrillary tangles in Alzheimer’s disease brains [1]. Since then, deposition of abnormal tau in the brain has been found to be involved in a spectrum of neurodegenerative diseases, collectively termed ‘tauopathies’. The tauopathies are divided into primary tauopathies, depending on the role of the tau protein. In primary tauopathies, which include frontotemporal dementia, Pick’s disease, progressive supranuclear palsy, and corticobasal degeneration [2], tau is the main driver of the pathology, while in secondary tauopathies, such as Alzheimer’s disease, tauopathy occurs as a result of other proteinopathies.

Tau regulates the self-assembly of tubulin into microtubules in neurons, helping to stabilize the axonal cytoskeleton. This physiological process is dynamic and is modulated by the phosphorylation state of tau. The leading hypothesis for the development of tau pathology in Alzheimer’s disease is that it is a downstream event of amyloid beta (Aβ) plaque pathology, and that abnormal phosphorylation of tau causes the protein to detach from the microtubules, thereby destabilizing them, leading to axonal degeneration and the aberrant aggregation of tau into paired helical filaments, which in turn assemble into neurofibrillary tangles [3]. Many parts of this process are still unknown, including the initial upstream trigger of abnormal phosphorylation of tau and whether different phosphorylation sites have different pathophysiological roles.

Together with the Aβ42/40 ratio (an amyloid plaque biomarker), cerebrospinal fluid (CSF) tau has an important role as an Alzheimer’s disease biomarker. The concentrations of both pTau and non-phosphorylated tau, or “total” tau (tTau) are increased in Alzheimer’s disease patients [4]. While CSF pTau and tTau correlate tightly within Alzheimer’s disease and control cohorts, elevated CSF tTau, reflecting neurodegeneration, also occurs in other neurological conditions, such as Creutzfeldt-Jakob’s disease [5] and stroke [6], but increased pTau seems to be more specific to Alzheimer’s disease [4].

To date, CSF pTau has most frequently been measured by immunoassays that detect phosphorylation at amino acid Thr-181 (pT181), located in the mid-region of the protein, but methods to quantify other phospho-epitopes have also been evaluated, including pS199, pT217, and pT231, as well as the C-terminal residues pS396 and pS404 [7–13]. Recently, a study showed improved biomarker performance of pT217 compared with pT181 in Alzheimer’s disease [14]. However, two studies that compared pT181 and pT217 head-to-head in CSF, using the same N-terminal antibody (Tau12) as the detector in the two assays, suggested identical diagnostic performance of these two pTau variants [15, 16]. Yet, each of the N-terminal-directed pT181 and pT217 biomarkers became abnormal earlier in the disease process than standard mid-region pT181 biomarkers. Another study that used mass spectrometry to measure the degree of phosphorylation at different sites of tau in patients with dominantly inherited Alzheimer’s disease found that abnormal phosphorylation at Thr-217 and Thr-181 occurred significantly earlier than at Thr-205, beginning as early as 20 years before the onset of symptoms [17]. Furthermore, other studies have shown that CSF pT231 is a marker of incipient sporadic frontotemporal dementia pathophysiology, identifying early disease changes better than pT181 and pT217 biomarkers [16]. Additionally, pT396, an integral component of brain neurofibrillary tangles [18], has been shown to be increased in the CSF in Alzheimer’s disease patients compared with controls [9]. These studies suggest that the temporal sequence of tau phosphorylation at different epitopes provides disease-relevant insights and underscores the importance of further studying the phosphorylation at specific sites in tau.

Advances in antibody-based immunoassay technologies with improved sensitivity have enabled the quantification of very low abundant tau species also in other biofluids than CSF [14, 16, 19–22]. However, these platforms have some drawbacks. For example, multiplexed biomarker measurements are currently not available for pTau. Therefore, samples are analysed independently for each individual marker. Further, given that both tau (both tTau and pTau variants) are truncated into N-terminal to mid-domain fragments before being secreted to CSF and plasma [12], the signal in sandwich immunoassays will depend not only on which specific pTau species is captured, but also on which fragment of tau the pTau epitope is present.

Mass spectrometry (MS) has been instrumental in characterizing tau. In brain tissue, in which tau is abundant, over 80 utilized phosphorylation sites have been identified [23–25]. In CSF, the tau concentration is relatively low; 80 – 450 pg/ml in healthy individuals [26], and the proportion of pTau is roughly only 10% of tTau, making the phosphorylated forms challenging to detect against the background of higher-abundant CSF proteins. To date, this could only be achieved by using immunoprecipitation (IP) to enrich tau prior to liquid chromatography (LC)-MS [27]. While such IP-MS based methods can perform relative quantification, e.g., measuring the ratio of phosphorylated to non-phosphorylated peptide, provided that antibodies are available that binds both forms with equal affinity [28], absolute quantification may be biased as the measurement is affected by the affinity of the antibodies for the target protein. Using full-length isotope-labelled protein as internal standard can overcome this problem provided that the protein standard is captured by the antibody to the same extent as the endogenous protein.

In the present study, we present the first antibody-free method for quantification of phosphorylated tau epitopes in CSF. The method is based on parallel reaction monitoring (PRM) assay on a high-resolution orbitrap hybrid mass spectrometer and measures a multiplex panel of the phospho-epitopes pT181, pS199, pS202, pT205, pT217, pT231 and pS396, as well as two non-phosphorylated peptides covering amino acids 195–209 and 212–221. Stable-isotope labeled peptide calibrants are spiked directly into the neat CSF samples and co-purified by precipitation of other sample proteins using perchloric acid, followed by solid-phase extraction and LC–MS. Compared to IP-based methods, this method affords higher throughput and is more cost-effective. Importantly, as has previously been demonstrated, this sample preparation allows for absolute quantification of perchloric acid-soluble tau [29, 30]. This makes the novel method a suitable candidate to evaluate for the future development of reference methods and the detailed evaluation of pTau species in clinical routine and therapeutic response.

We evaluated the performance of the method by the analysis of clinical samples with Alzheimer’s disease—and non- Alzheimer’s disease biomarker profiles and compared the results with those of pTau immunoassays developed on the Single molecule array (Simoa) platform, and then used the method to analyze a cohort comprising the entire Alzheimer’s disease spectrum.

## Materials and methods

### Study populations

A pilot study was performed of CSF samples submitted to the Clinical Routine Laboratory at the Sahlgrenska University Hospital, Mölndal, Sweden, that had previously been assayed for the core frontotemporal dementia biomarkers, measured by Lumipulse G Amyloid AMYLOID (1‐42), PHOSPHO‐TAU (181P), and hTAU immunoassays from FujiRebio Europe (Antwerp, Belgium) according to the manufacturers’ instructions. Samples with tTau > 440 pg/ml, pTau > 61 pg/ml, and amyloid-β 1–42 < 620 pg/ml were classified as Alzheimer’s disease-type, and samples with normal biomarker profile were assigned as controls.

The second set of samples consisted of cross-sectional CSF samples from the Translational Biomarkers in Aging and Dementia (TRIAD) cohort. The TRIAD includes participants within the whole Alzheimer’s disease spectrum which were highly profiled with clinical and neuropsychological assessments as well as with fluid and imaging biomarkers. In this cohort, the Alzheimer’s disease dementia diagnosis was given following the National Institute on Aging and the Alzheimer’s Association criteria for probable Alzheimer’s disease [31], with a Clinical Dementia Rating (CDR) greater than 1. MCI patients had CDR of 0.5, subjective and objective memory impairments but essentially normal activities of daily living. CU individuals had CDR of 0. Participants clinically diagnosed with frontotemporal dementia (clinical diagnosis of behavioral or semantic variant of frontotemporal dementia, CDR score > 0.5 and Aβ positron emission tomography (PET) negative) were also included. For this analysis, were included only participants that had CSF samples available at the time of the experiment and PET imaging data available at the time of the analysis. In addition, participants were re-classified according to clinical diagnosis and Aβ status (positive ( +) and negative (-)) into young cognitively unimpaired (CU) Aβ-participants (*n*_Young_ = 22), elderly CU Aβ- (*n*_CU-_ = 54), elderly CU Aβ + (*n*_CU+_  = 26), MCI Aβ + (*n*_MCI+_  = 19), Alzheimer’s disease Aβ + (*n*_AD_ = 19), MCI and Alzheimer’s disease Aβ- (*n*_non-AD_ = 16) or frontotemporal dementia (*n*_FTD_ = 9).

As a third cohort, samples from the prospective Swedish BioFINDER-2 cohort were analyzed [32], which included patients with mild cognitive impairment (MCI), Alzheimer’s disease with dementia, and a spectrum of other neurodegenerative diseases, as well as cognitively unimpaired (CU) controls. The patients with Alzheimer’s disease fulfilled the Diagnostic and Statistical Manual of Mental Disorders [Fifth Edition] Alzheimer’s disease criteria [33] and were required to be Aβ-positive. Further subdivision into Aβ-positive/negative participants of the cognitively unimpaired participants and participants with MCI was performed as well as into preclinical Alzheimer’s disease and Alzheimer’s disease with MCI if they were Aβ-positive and tau-positive participants without cognitive impairment and with MCI, respectively [34]. Inclusion criteria for the other neurodegenerative diseases included fulfillment of criteria for frontotemporal dementia [33], Parkinson’s disease [35], PD with dementia [33], subcortical vascular dementia [33], progressive supranuclear palsy [36], multiple system atrophy [37], or semantic variant primary progressive aphasia [38]. The CU participants were required to not the fulfil criteria for mild cognitive impairment or dementia, including having no history of cognitive change over time and having a Clinical Dementia Rating score of 0. Participants were recruited at Skåne University Hospital between April 2017 to September 2019. All participants underwent the Mini-Mental State Examination to assess global cognition [39]. Ethical approval was given by the Regional Ethical Committee in Lund, Sweden. Demographics are shown in Table 1 and for full details on diagnostic criteria, refer to [40].

**Table 1:** Demographic and biomarker information of the TRIAD cohort by clinical and biomarker-defined groups

	**Young adults (*n* = 22)**	**CU-** **(*n* = 54)**	**CU + ** **(*n* = 26)**	**MCI + ** **(*n* = 19)**	**AD** **(*n* = 19)**	**Non-AD** **(*n* = 16)**	**FTD** **(*n* = 9)**	***P*-value**
Age, years	23.3 (1.8)	71.0 (7.4)	72.2 (7.5)	72.2 (6.1)	64.6 (7.1)	70.5 (9.5)	62.4 (6.2)	< 0.0001*
Female, n (%)	14 (63)	34 (63)	17 (65)	9 (47)	8 (42)	7 (43)	6 (66)	0.44
Education, years	16.7 (1.5)	14.9 (3.6)	14.4 (2.7)	15.8 (2.8)	15.4 (3.1)	13.7 (4.0)	14.4 (4.0)	0.08
*APOE*-ε4 carriers, n (%)	6 (27)	17 (31)	8 (30)	12 (63)	13 (72)	4 (26)	1 (11)	0.002*
MMSE	29.7 (0.5)	29.1 (1.0)	29.3 (0.8)	27.9 (1.8)	20.0 (6.3)	26.7 (2.8)	26.0 (7.3)	< 0.0001*
Aß PET (SUVR)	1.18 (0.06)	1.26 (0.10)	1.96 (0.42)	2.40 (0.42)	2.35 (0.44)	1.33 (0.12)	1.15 (0.08)	< 0.0001*
Tau PET (SUVR)	0.82 (0.08)	0.91 (0.10)	1.00 (0.15)	1.56 (0.45)	2.09 (0.55)	1.11 (0.65)	0.79 (0.10)	< 0.0001*
p-Tau181 (fmol/mL)	0.86 (0.25)	1.15 (0.33)	1.41 (0.49)	1.79 (0.71)	1.74 (0.69)	1.48 (0.95)	0.98 (0.43)	< 0.0001*
p-Tau199 (fmol/mL)	0.17 (0.06)	0.24 (0.05)	0.29 (0.08)	0.35 (0.12)	0.43 (0.17)	0.32 (0.24)	0.24 (0.07)	< 0.0001*
p-Tau202 (fmol/mL)	0.06 (0.02)	0.09 (0.03)	0.10 (0.03)	0.13 (0.06)	0.15 (0.05)	0.12 (0.06)	0.09 (0.04)	< 0.0001*
p-Tau205 (fmol/mL)	0.01 (0.004)	0.02 (0.01)	0.03 (0.01)	0.08 (0.07)	0.11 (0.05)	0.05 (0.09)	0.02 (0.009)	< 0.0001*
p-Tau217 (fmol/mL)	0.04 (0.01)	0.08 (0.04)	0.20 (0.13)	0.40 (0.25)	0.54 (0.31)	0.25 (0.43)	0.12 (0.17)	< 0.0001*
p-Tau231 (fmol/mL)	0.02 (0.02)	0.07 (0.07)	0.29 (0.29)	0.50 (0.26)	0.60 (0.38)	0.31 (0.53)	0.12 (0.24)	< 0.0001*
p-Tau396 (fmol/mL)	0.03 (0.01)	0.05 (0.02)	0.06 (0.03)	0.07 (0.02)	0.06 (0.03)	0.06 (0.03)	0.04 (0.02)	< 0.0001*

Data shown as mean (SD) or n (%), as appropriate. Variables were compared with a one-way ANOVA adjusted and *P*-values are presented. Aβ status for group definition was based on PET visual rating

*Abbreviations*: *Aβ* amyloid-β, *AD* Alzheimer’s Disease dementia, *CU-* Aβ-negative cognitively unimpaired, *CU* + Aβ-positive cognitively unimpaired, *FTD* Aβ- Fronto-temporal dementia, *MCI* + Aβ-positive mild cognitive impairment, *MMSE* Mini-Mental State Examination, *Non-AD* Aβ-negative MCI and “AD” dementia, *SUVR* Standardized uptake value ratio

### Sample preparation for LC–MS analysis of pTau epitopes in CSF

CSF samples (250 µl) were spiked with 10 µl of a mixture of heavy isotope-labeled peptide standards (AQUA peptides, Thermo Scientific). The spike-in amount of each heavy peptide was adjusted to yield a light-to-heavy peak area ratio of approx. 0.1 – 0.2 in CSF from non-AD subjects (Supplementary Table S1). The peptide standards were diluted from 10 pmol lyophilized aliquots in 20% acetonitrile, with the final 1:10 dilution step performed in 50 mM ammonium bicarbonate to avoid potential interference from acetonitrile in the sample preparation. Protein precipitation was performed by adding perchloric acid (15 µl, 60% v/v) to the samples, which then were briefly vortexed and incubated on ice for 15 min. Under these conditions, a majority of CSF proteins precipitate, but not tau. The precipitated proteins were pelleted by centrifugation at 30,000 × g for 10 min at 4 °C, and the supernatants were transferred to a 96-well filter microtitre plate (AcroPrep Advance, 350 µl, 0.45 µm, Supor membrane, Pall Corporation). A vacuum manifold was used to pass the samples through the filter plate, and directly load them on a 96-well SPE plate (Oasis PRiME HLB 96-well µElution Plate, 3 mg Sorbent per Well, Waters). The SPE plate was washed twice with 200 µl 5% methanol (v/v), and peptides were eluted into a microtitre plate with 200 µl 50% acetonitrile, 0.1% trifluoroacetic acid, and the eluates were lyophilized by vacuum centrifugation. Trypsin (Sequencing grade, Promega) was dissolved in the diluent provided by the manufacturer and diluted to 2.5 µg/ml in 50 mM ammonium bicarbonate. Trypsin solution (40 µl) was added to the dry samples, which were vortexed and incubated at 37 °C overnight. TFA (1 µl, 10% v/v) was added to the samples to quench further proteolysis. The samples were stored at -20 °C prior to LC–MS analysis.

### LC–MS

The samples were analyzed by LC–MS on a Ultimate 3000 nanoflow-LC (RSLC nano, Thermo Scientific) equipped with a trap column (300 μm i.d. × 5 mm packed with Acclaim PepMap 100 C18, 5 μm, Thermo Scientific), and a separation column (Easy Spray 75 μm × 500 mm, C18, 2 μm, 100 Å, Thermo Scientific), coupled to a hybrid Orbitrap mass spectrometer (Fusion Tribrid, Thermo Scientific), fitted with a EasySpray nano-ESI ion source. The loading buffer was 0.05% TFA, Buffer A was 0.1% formic acid (v/v), and Buffer B was 84% acetonitrile (v/v), 0.1% (v/v) formic acid. The loading pump was operated at 50 µl/min. After loading samples on the trap column (5 min), the trap was switched in line with the separation column and the following gradient was applied using the nano-flow pump: *t* = 0 min, B = 5%; *t* = 5 min, B = 5%; *t* = 30 min, B = 30%; *t* = 30.5, B = 100%; *t* = 40 min, B = 100%; *t* = 40.5 min, B = 5%; *t* = 50 min, B = 5%. The mass spectrometer was operated in the positive ion mode, with the following settings for the PRM scan: Activation Type: HCD; Collision energies were determined experimentally for each peptide and are listed in Supplementary Table S1; Detector Type: Orbitrap; Orbitrap Resolution: 120 000; Scan Range: 150–1500; RF Lens: 60%; Easy-IC: On; Isolation Type: Quadrupole; Isolation Window: 1.2 m*/z*; Maximum Injection Time: 400 ms; Normalized AGC Target: 1000%. LC–MS data was analyzed using the software Skyline v. 21 (McCoss Lab, University of Washington).

### Simoa immunoassays

CSF samples from the discovery and validation cohorts were analysed using immunoassays for tau pT181, pT217 and pT231 on the Single molecule array (Simoa) platform as previously [15, 16, 41]. For each biomarker, two or three internal quality control samples were measured at the start and the end of each analytical run. For all three analytes, the within-plate coefficients of variation (CV) were ≤ 1.7 and 0.2% respectively. All samples measured above the respective assay lower limits of detection.

### Imaging analysis

Brain Aβ and tau load were indexed based on PET imaging, using [^18^F]AZD4694 and [^18^F]MK6240 respectively. All participants underwent 3 T T1-weighted images for co-registration purposes. PET imaging acquisition was performed on Siemens High Resolution Research and scans were reconstructed using the ordered subset expectation maximization (OSEM) algorithm on a 4-dimensional volume as previously described (PMID: 22,323,782, PMID: 30,064,520, PMID: 31,860,000). The reference regions were the inferior cerebellum and the whole cerebellum gray matter for [^18^F]MK6240 and [^18^F]AZD4694, respectively. Global Aβ PET positivity was visually defined by two neurologists blinded to clinical diagnosis. Tau PET Braak stage classification was defined as described in a previous study [42].

### Statistical analysis

Statistical analyses were performed on R v3.6.3 (http://www.R-project.org/) and tests were 2-tailed, with α = 0.05. The normality of the biomarker distribution was visually assessed by the inspection of histograms and Q-Q plots and, when required, variables were log10 transformed to assure normal data distribution. Linear regression models compared the biomarker distribution (log-transformed) across groups, covariating for age and sex. When post hoc analysis was needed, Tukey honestly significant difference (HSD) was employed. Correlations between variables were assessed using Spearman rank correlation test. Fold changes compared the mean of the biomarkers in the Alzheimer’s disease dementia group with the mean of the CU- group. Using the generalized linear model (GLM) binary regression framework receiver operating curves (ROC) were estimated, which provided the area under the curve (AUC) for Aβ positivity, or diagnostic groups, for each biomarker. The two biomarkers with the greatest AUC were compared with the “*pROC*” package.

### Data availability

The authors confirm that the data supporting the findings of this study are available within the article and its Supplementary material.

## Results

### Antibody-free measurement of tau phosphorylation in CSF

To achieve sufficient CSF sample clean-up to enable nano-LC–MS analysis without using antibody-based immunoprecipitation, a sample preparation protocol was developed that is based on partial protein precipitation using perchloric acid, followed by reversed-phase SPE in the 96-well format, and finally trypsin digestion. Neat CSF samples were spiked with a mixture of heavy isotope-labeled peptide standards with amino acid sequences matching the trypsin proteolysis products containing the targeted phosphorylation sites (Supplementary Table S1). A set of *b*- and *y*-ions, clearly detectable and free from interfering signals from the CSF sample matrix were used for quantification. Representative examples of PRM chromatograms are shown in Supplementary Figure S1.

pS199, pS202, and pT205 were detected on isobaric tryptic peptides with identical amino acid sequence. pS199 (Supplementary Figure S1 B) and pS202 (Supplementary Figure S1 C) co-eluted in the LC separation but could be quantified separately by using distinct fragments ions formed by cleavage between the two phosphorylation sites (b_5-7_ and y_8-10_, and corresponding fragments formed by neutral loss of phosphoric acid (-98 Da)). pT205 was resolved chromatographically from the other two isobaric peptides, and thus all prominent fragment ions detected from it could be used for quantification (Supplementary Figure S1 D).

The reproducibility of the method was determined by the analysis of eight aliquots of a CSF pool (Supplementary Table S2). At the time of analysis, no heavy calibrator was available for pT205; instead, heavy pS199 peptide was used as calibrator. The lowest variation was observed for pS202 (*CV* = 3.1%) and the highest variation was observed for pT217 (*CV* = 19.01%). The CSF pool used for the measurements was composed of patient samples with a non-AD-type core biomarker profile, i.e., with low pTau and tTau levels. Thus, the low levels of the phosphopeptides are likely to contribute to the higher CVs observed for some of the peptides.

### Pilot study

The performance of the pTau PRM method was evaluated by analyzing CSF samples from patients with AD-indicative and normal neurochemical biomarker profiles (*n* = 38), based on the core Alzheimer’s disease biomarkers (Supplementary Figure S2, Supplementary Table S3). All pTau species showed high performance for identifying Alzheimer’s disease (AUC > 95%), with the largest fold-change (FC) observed for pT217 (FC = 7.7), followed by pT231 (FC = 6.7). pT217 also showed the largest effect size (Cohen’s *d* = 3.2), followed by pT205 (*d* = 2.5).

### Correlation with Simoa immunoassay measurements

The pTau PRM method was also compared with digital immunoassays for pT181, pT217, and pT231 on the Simoa platform, by measuring a second set of CSF samples from the Discovery cohort (*n* = 44) with Alzheimer’s disease and normal core CSF biomarker profiles. The PRM method showed strong correlation with the Simoa immunoassays for all three analytes (pT181: Spearman (*ρ*) = 0.95, pT217: *ρ* = 0.79, pT231: *ρ* = 0.94, Supplementary Figure S3). The PRM methods for pT181, pT217, and pT231 also strongly correlated with each other (*ρ* = 0.94–0.96).

### Tau phosphorylation across the Alzheimer’s disease continuum

To explore how the abundance of phosphorylated tau epitopes develop over the course of AD, CSF samples from two large cohorts were analyzed: TRIAD (*n* = 165) and BioFINDER-2 (*n* = 563). The patients in both cohorts had been subjected to clinical evaluation of cognitive function as well as measurement of Aβ and tau pathology by PET imaging. Demographic information, clinical diagnosis, and Aβ and tau PET status are presented in Table 1 and 2.

**Table 2:** Demographic and biomarker information of the BioFINDER-2 cohort by clinical and biomarker-defined groups

	**CU-** **(*n* = 236)**	**CU + ** **(*n* = 73)**	**MCI-** **(*n* = 44)**	**MCI + ** **(*n* = 68)**	**AD** **(*n* = 81)**	**Non-AD-** **(*n* = 33)**	**Non-AD + ** **(*n* = 28)**	***P*-value**
Age, years	60.8 (14.9)	70.7 (8.2)	68.7 (8.0)	72.4 (7.4)	73.5 (7.2)	72.6 (8.2)	74.7 (6.0)	< 0.0001*
Female, n (%)	118 (50)	31 (43)	15 (34)	35 (52)	41 (51)	9 (27)	10 (36)	0.074
Education, years ^a^	12.7 (3.1)	12.3 (3.8)	12.2 (3.6)	12.4 (4.1)	11.8 (3.9)	10.6 (3.4)	13.8 (4.0)	0.008*
*APOE*-ε4 carriers, n (%) ^b^	84 (35.6)	50 (68.5)	10 (22.7)	48 (70.6)	54 (66.7)	5 (15.2)	17 (60.7)	< 0.0001*
MMSE	28.7 (1.8)	28.6 (1.8)	27.5 (1.9)	26.7 (1.9)	19.8 (4.7)	22.7 (3.7)	21.8 (5.6)	< 0.0001*
Aß PET (SUVR)	0.62 (0.04)	0.84 (0.19)	0.63 (0.05)	0.98 (0.19)	1.08 (0.18)	0.63 (0.03)	-	< 0.0001*
Tau PET (SUVR)	1.13 (0.10)	1.29 (0.34)	1.16 (0.09)	1.44 (0.43)	2.12 (0.65)	1.18 (0.16)	1.31 (0.21)	< 0.0001*
p-Tau181 (fmol/mL)	0.94 (0.33)	1.13 (0.45)	0.97 (0.27)	1.49 (0.58)	1.76 (0.77)	1.07 (0.52)	1.17 (0.34)	< 0.0001*
p-Tau199 (fmol/mL)	0.20 (0.09)	0.26 (0.10)	0.23 (0.10)	0.30 (0.12)	0.39 (0.16)	0.25 (0.11)	0.26 (0.08)	< 0.0001*
p-Tau202 (fmol/mL)	0.06 (0.03)	0.08 (0.05)	0.07 (0.04)	0.08 (0.03)	0.10 (0.04)	0.06 (0.03)	0.07 (0.03)	< 0.0001*
p-Tau205 (fmol/mL)	0.02 (0.03)	0.04 (0.04)	0.02 (0.02)	0.05 (0.04)	0.09 (0.06)	0.02 (0.02)	0.03 (0.02)	< 0.001*
p-Tau217 (fmol/mL)	0.06 (0.04)	0.21 (0.17)	0.08 (0.06)	0.31 (0.22)	0.57 (0.32)	0.10 (0.09)	0.18 (0.08)	< 0.0001*
p-Tau231 (fmol/mL)	0.02 (0.03)	0.07 (0.11)	0.02 (0.02)	0.10 (0.14)	0.20 (0.28)	0.04 (0.05)	0.04 (0.05)	< 0.0001*
p-Tau396 (fmol/mL)	0.06 (0.11)	0.07 (0.06)	0.05 (0.04)	0.09 (0.10)	0.08 (0.06)	0.05 (0.04)	0.04 (0.02)	0.038*

Data shown as mean (SD) or n (%), as appropriate. Variables were compared with a one-way ANOVA adjusted and *P*-values are presented. Aβ status for group definition was based on CSF Aβ levels *Abbreviations*: *Aβ* Amyloid-β, *AD* Alzheimer’s Disease dementia, *CU-* Aβ-negative cognitively unimpaired, *CU* + Aβ-positive cognitively unimpaired, *MCI-* Aβ-negative mild cognitive impairment, *MCI* + Aβ-positive mild cognitive impairment, *MMSE* Mini-Mental State Examination, *Non-AD-* Aβ-negative and non-AD dementia, *Non-AD* + Aβ-positive and non-AD dementia, *SUVR* Standardized uptake value ratio

^a^ Data is missing for two individuals

^b^ Data is missing for three individuals

The average age of the TRIAD study population was 63.7 years, 57% were females and 36% were *APOE*-ε4 carriers. As expected, the Alzheimer’s disease dementia group had lower average MMSE scores, as well as higher frequency of *APOE*-ε4 carriers as compared to MCI + , non-AD and the CU groups. The average age of the BioFINDER-2 study population was 67.3 years, 46% were females and 48% were *APOE*-ε4 carriers. Also in BioFINDER-2, the Alzheimer’s disease dementia group had lower average MMSE scores and higher frequency of *APOE*-ε4 carriers compared to MCI + , non-AD and the CU groups.

**Fig. 1:**
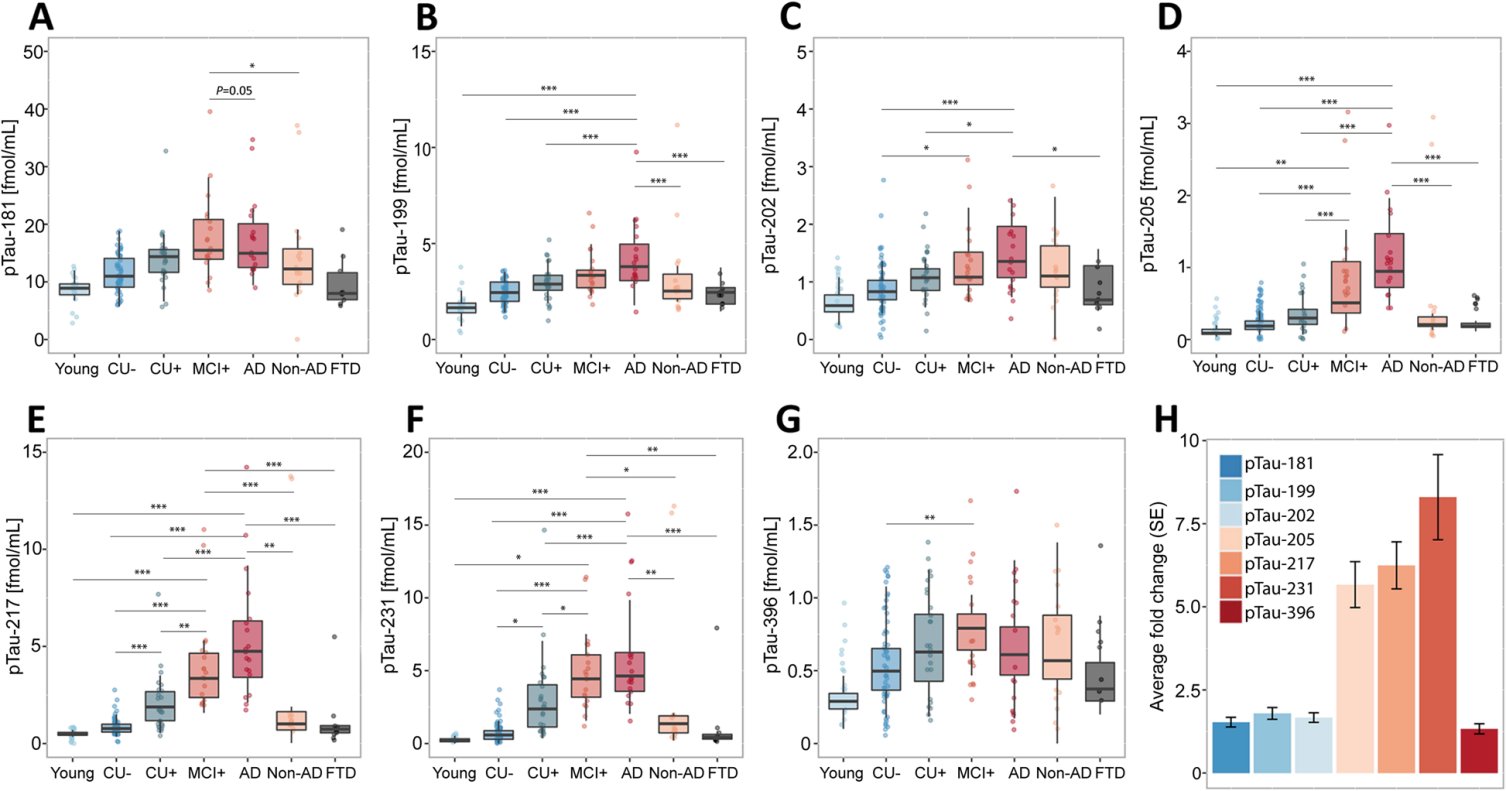
Abundances of phosphorylated tau epitopes across the Alzheimer’s disease spectrum in the TRIAD cohort (**A**-**G**). The concentrations of the seven phosphorylated tau epitopes, pTau-181 (**A**), pTau-199 (**B**), pTau-202 (**C**), pTau-205 (**D**), pTau-217 (**E**), pTau-231 (**F**) and pTau-396 (**G**) are plotted for the different groups. The boxplots depict the median (horizontal bar), interquartile range (IQR, hinges) and 1.5 × IQR (whiskers). Group comparisons were computed with a one-way ANCOVA adjusting for age and sex. Tukey honestly significant difference test was used for the post hoc pairwise comparisons in all cohorts. Biomarker fold change between CU- and Alzheimer’s disease groups (H). Abbreviations: Aβ, amyloid-β, AD, Alzheimer’s disease; CU-, Aβ-negative cognitively unimpaired; CU + , Aβ-positive cognitively unimpaired; frontotemporal dementia; MCI + , Aβ-positive mild cognitive impairment; Non-AD, Aβ-negative “AD” dementia or mild cognitive impairment patients. ^*^P < 0.05; ^**^P < 0.01, ^***^P < 0.001

**Fig. 2:**
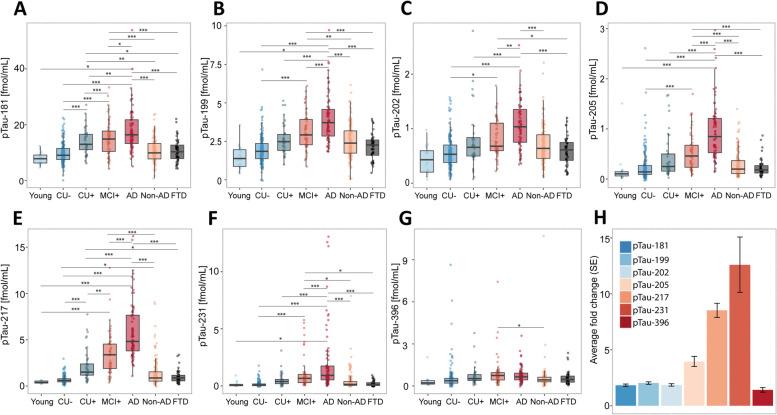
Abundances of phosphorylated tau epitopes across the Alzheimer’s disease spectrum in the BioFINDER-2 cohort (**A**-**G**). The concentrations of the seven phosphorylated tau epitopes, pTau-181 (**A**), pTau-199 (**B**), pTau-202 (**C**), pTau-205 (**D**), pTau-217 (**E**), pTau-231 (**F**) and pTau-396 (**G**) are plotted for the different groups. The boxplots depict the median (horizontal bar), interquartile range (IQR, hinges) and 1.5 × IQR (whiskers). Group comparisons were computed with a one-way ANCOVA adjusting for age and sex. Tukey honestly significant difference (HSD) test was used for the post hoc pairwise comparisons in all cohorts. Biomarker fold change, presented in (**H**) between CU- and Alzheimer’s disease groups. Abbreviations: Aβ, amyloid-β, AD, Alzheimer’s disease; CU-, Aβ-negative cognitively unimpaired; CU + , Aβ-positive cognitively unimpaired; MCI + , Aβ-positive mild cognitive impairment; Non-AD, Aβ-negative “AD” dementia or mild cognitive impairment patients; Other. ^*^P < 0.05; ^**^P < 0.01, ^***^P < 0.001

To explore how the abundances of phosphorylated tau epitopes change over the course of AD, the study participants of both cohorts were organized by clinical assessment and PET data to form an Alzheimer’s disease continuum (Fig. 1 A-G and Fig. 2 A-G), starting with young, healthy individuals, followed by cognitively unimpaired elderly without Aβ pathology (CU-), proceeding to cognitively unimpaired with Aβ pathology (CU +), symptomatic elderly with mild cognitive impairment and Aβ pathology (MCI +), and ending with clinical AD, verified by Aβ-PET or CSF Aβ1-42/1–40 ratio. On this continuum, all measured pTau epitopes increased in both cohorts, with the best distinction between the different disease stages and the CU- group observed for pT231 (TRIAD: AUC = 93.61%, fold change = 5.72; BioFINDER-2: AUC = 84.44, fold change = 6.40), pT217 (TRIAD: AUC = 92.8%, fold change = 4.26), and pT205 (AUC = 85.07%, fold change = 3.35, Supplementary Table S4 and Supplementary Table S5, Supplementary Figure S4 and Supplementary Figure S5). To distinguish between Alzheimer’s disease and CU-, the best performance was observed for pT217 (TRIAD: AUC = 99.71%, fold change = 6.33; BioFINDER-2: AUC = 98.12%, fold change = 8.83), pT231 TRIAD: AUC = 98.73%, fold change = 7.64; BioFINDER-2: AUC = 91.89%, fold change = 10.65), and pT205 (TRIAD AUC = 99.07%, fold change = 5.34; BioFINDER-2: AUC = 93.51%, fold change = 3.92). These three peptides were also significantly increased in MCI + compared with CU- (pT217, TRIAD: AUC = 98.93%, fold change = 4.73; BioFINDER-2: AUC = 94.79%, fold change = 4.86), pT231, TRIAD: AUC = 98.54%, fold change = 6.44), and between CU + and CU- (pT217, TRIAD: AUC = 83.26%, fold change = 2.39; BioFINDER-2: AUC = 91.05, fold change = 3.29). While pT205 and pT217 increased further from MCI + to Alzheimer’s disease dementia, pT231 plateaued at MCI + . Non-AD participants as well as frontotemporal dementia (TRIAD), and patients suffering from other dementias (BioFINDER-2) showed biomarker levels comparable to the CU- group for all phospho-epitopes in both cohorts. Group differences were less marked for pT181, pS199 and pS202 and no significant group differences were observed for pS396 levels. The largest fold-changes between Alzheimer’s disease and CU- were also observed for, pT231, pT217 and pT205 in both cohorts (Fig. 1 H and 2 H), with pT231 showing a 5.6-fold increase in TRIAD and a 6.4-fold increase in BioFINDER-2. pS199, pS202, and pS396 showed a lower and similar increase of approximately 1.5-fold.

For the phosphorylated tau peptides for which it was possible to also measure the corresponding non-phosphorylated peptide (pS199, pS202, pT205 and pT217), we also calculated the ratios of phosphorylated to non-phosphorylated epitope (Supplementary Figure S6). We found that for all phospho-epitopes, the fold changes in Alzheimer’s disease versus CU- cases were smaller for the phospho-epitope ratios compared with the concentrations of the phospho-epitopes by themselves, quantified using the isotope-labelled internal standards.

### Correlation of pTau forms with Aβ PET

**Fig. 3:**
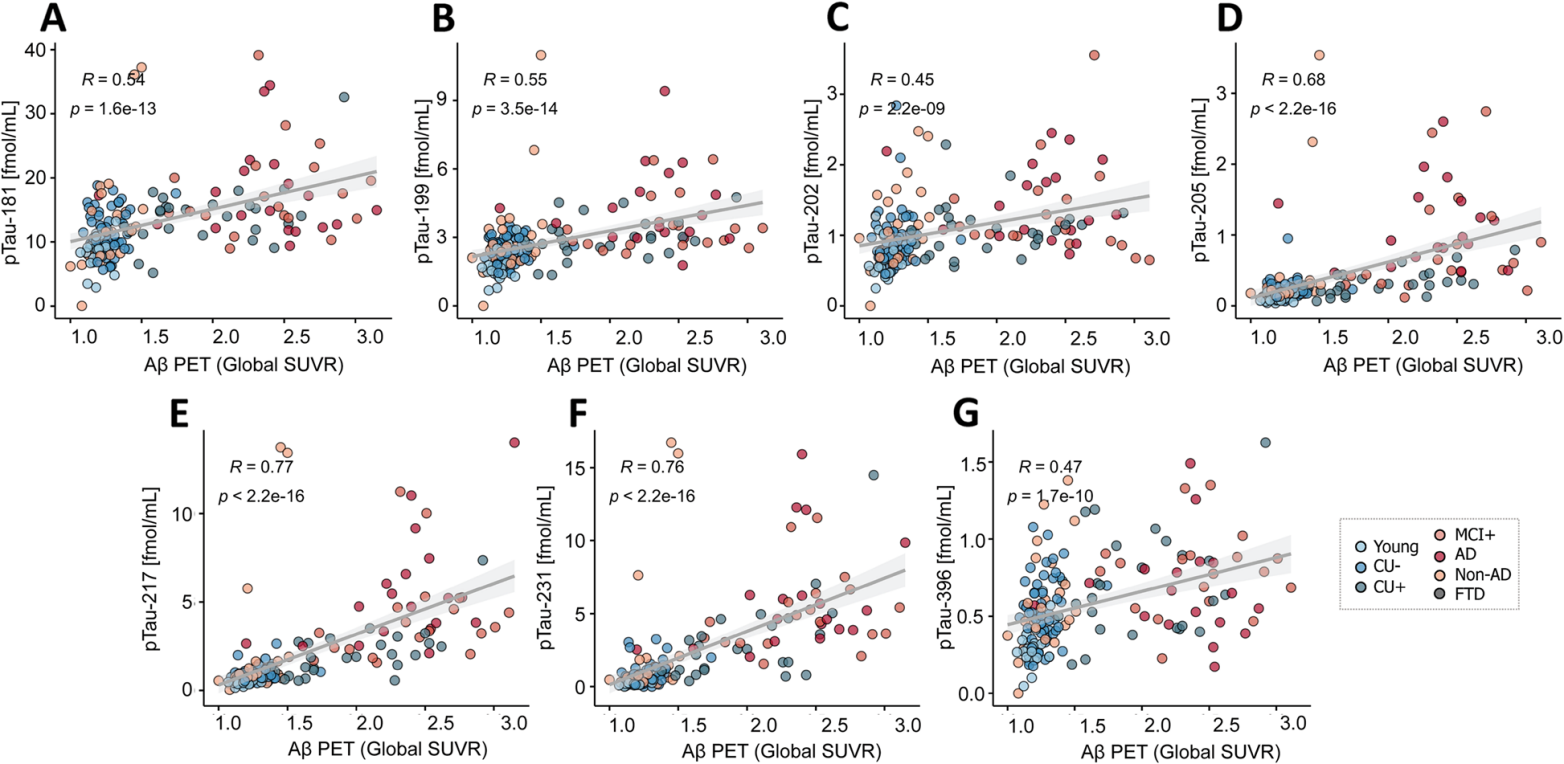
Correlation between the pTau biomarkers and Aβ PET indicated by Aβ PET (global average SUVR) in the TRIAD cohort, for pTau-181 (**A**), pTau-199 (**B**), pTau-202 (**C**), pTau-205 (**D**), pTau-217 (**E**), pTau-231 (**F**) and pTau-396 (**G**). Abbreviations: Aβ, amyloid-β; AD, Alzheimer’s disease; CU-, Aβ-negative cognitively unimpaired; CU + , Aβ-positive cognitively unimpaired; frontotemporal dementia; MCI + , Aβ-positive mild cognitive impairment; Non-AD, Aβ-negative “AD” dementia or mild cognitive impairment patients; *P*, *P* value of the correlation test; PET, positron emission tomography; *ρ*, Spearman rank correlation coefficient; SUVR, standard uptake value ratio

**Fig. 4:**
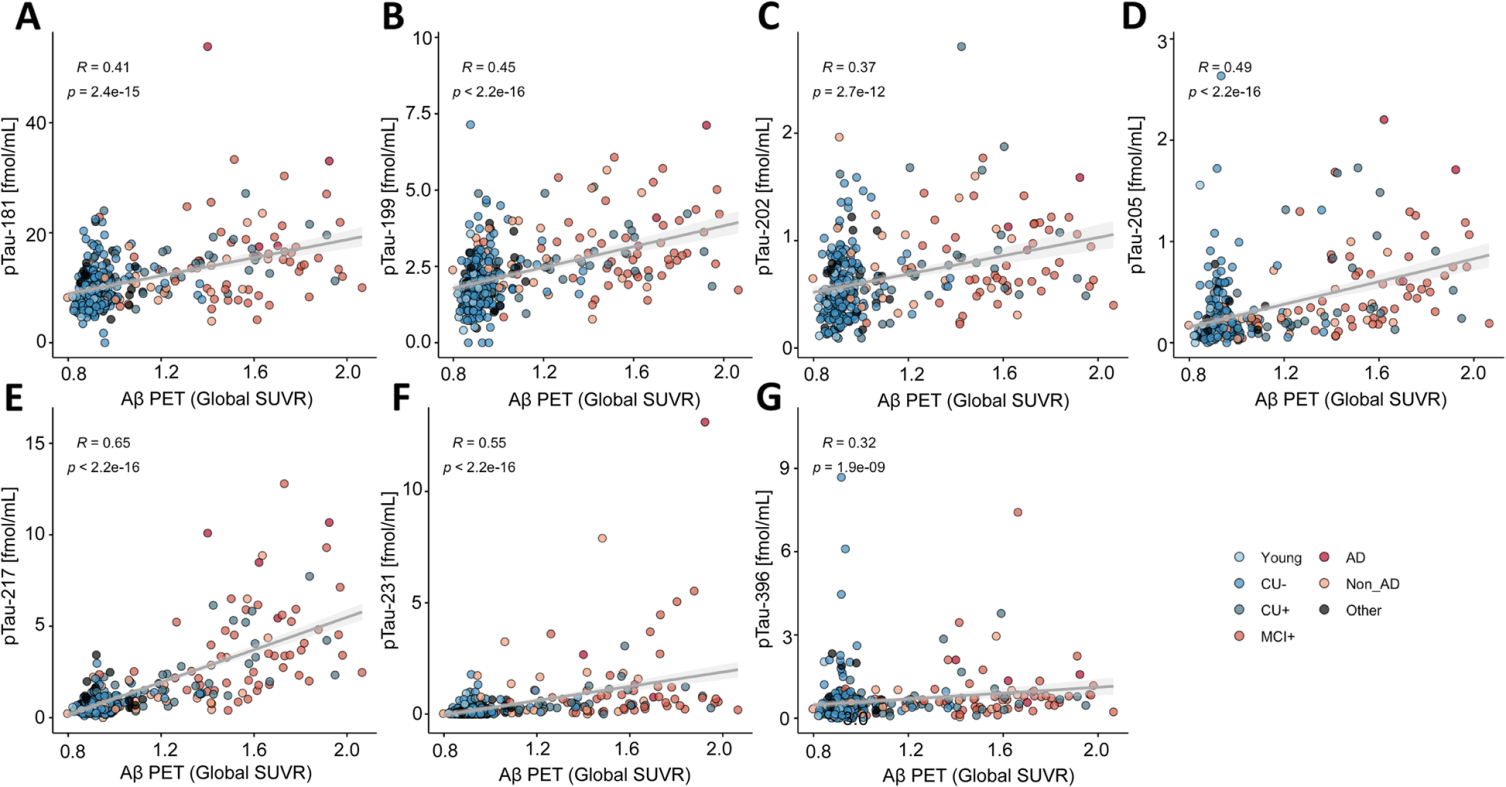
Correlation between the pTau biomarkers and Aβ PET (global average SUVR) in the BioFinder cohort, for pTau-181 (**A**), pTau-199 (**B**), pTau-202 (**C**), pTau-205 (**D**), pTau-217 (**E**), pTau-231 (**F**) and pTau-396 (**G**). Abbreviations: Aβ, amyloid-β; AD, Alzheimer’s disease; CU-, Aβ-negative cognitively unimpaired; CU + , Aβ-positive cognitively unimpaired; frontotemporal dementia; MCI + , Aβ-positive mild cognitive impairment; Non-AD, Aβ-negative “AD” dementia or mild cognitive impairment patients; *P*, *P* value of the correlation test; PET, positron emission tomography; *ρ*, Spearman rank correlation coefficient; SUVR, standard uptake value ratio

Analysis of the correlations between the abundances of the different pTau epitopes with the average standardized uptake values ratio (SUVR) of Aβ PET showed the strongest correlation for pT217, pT231 and pT205, both in TRIAD (Spearman (*ρ*)_pTau-217_ = 0.77, *P*_pTau-217_ < 0.001; *ρ*_pTau-231_ = 0.76, *P*_pTau-231_ < 0.001; *ρ*_pTau-205_ = 0.68, *P*_pTau-205_ < 0.001; Fig. 3) and BioFINDER-2 ((*ρ*)_pTau-217_ = 0.65, *P*_pTau-217_ < 2.2*10^–16^; *ρ*_pTau-231_ = 0.55, *P*_pTau-231_ < 2.2*10^–16^; *ρ*_pTau-205_ = 0.49, *P*_pTau-205<_2.2*10^–16^; Fig. 4).

### Correlation of pTau forms with Tau PET

**Fig. 5:**
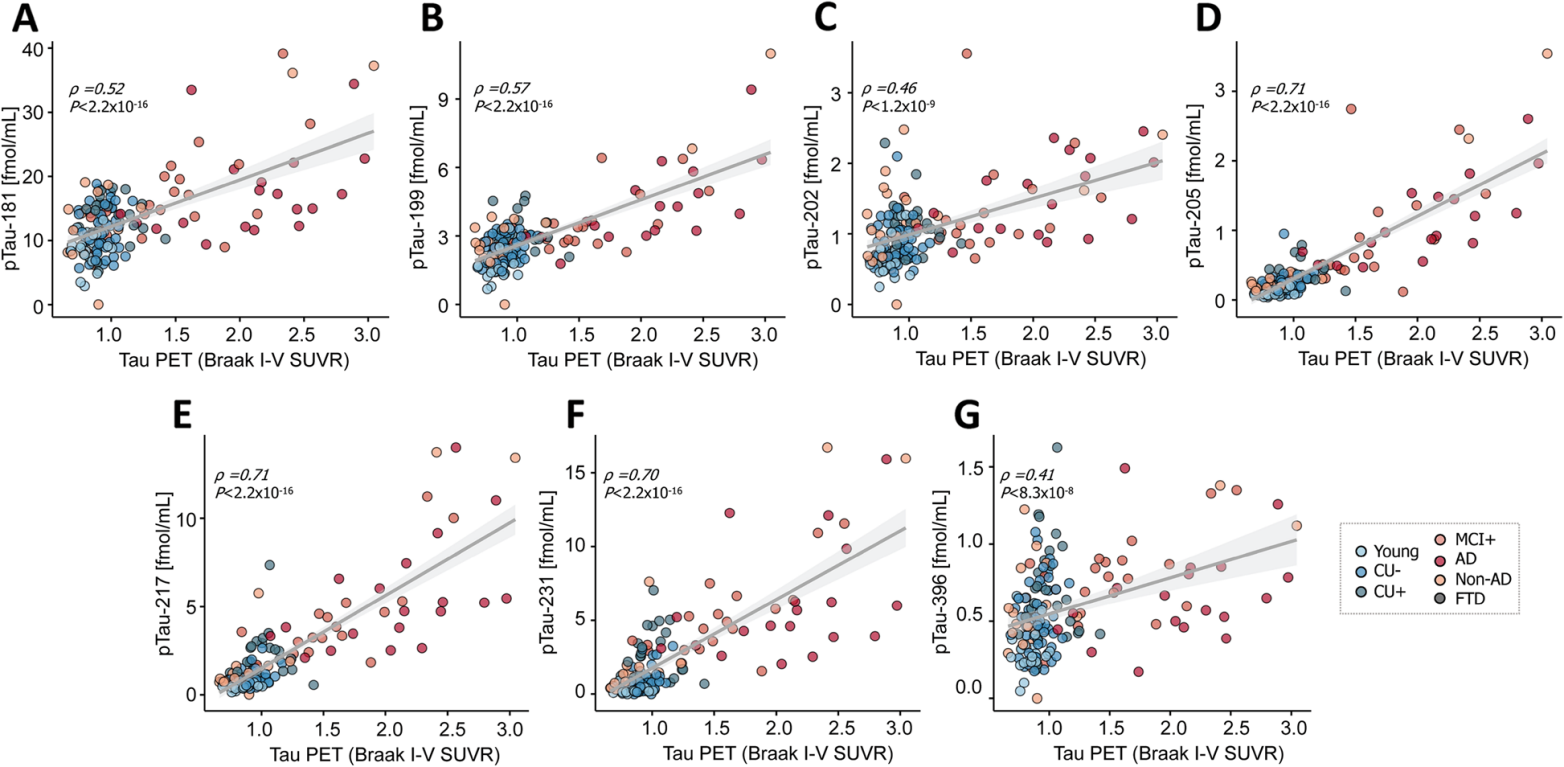
Correlation between the pTau biomarkers and tau PET (average SUVR of the regions corresponding to Braak stages I-IV) in the TRIAD cohort, for pTau-181 (**A**), pTau-199 (**B**), pTau-202 (**C**), pTau-205 (**D**), pTau-217 (**E**), pTau-231 (**F**) and pTau-396 (**G**) and tau pathology. Abbreviations: Aβ, amyloid-β; AD, Alzheimer’s disease; CU-, Aβ-negative cognitively unimpaired; CU + , Aβ-positive cognitively unimpaired; frontotemporal dementia; MCI + , Aβ-positive mild cognitive impairment; Non-AD, Aβ-negative “AD” dementia or mild cognitive impairment patients; p, *P* value of the correlation test; PET, positron emission tomography; R, Spearman rank correlation coefficient; SUVR, standard uptake value ratio

**Fig. 6:**
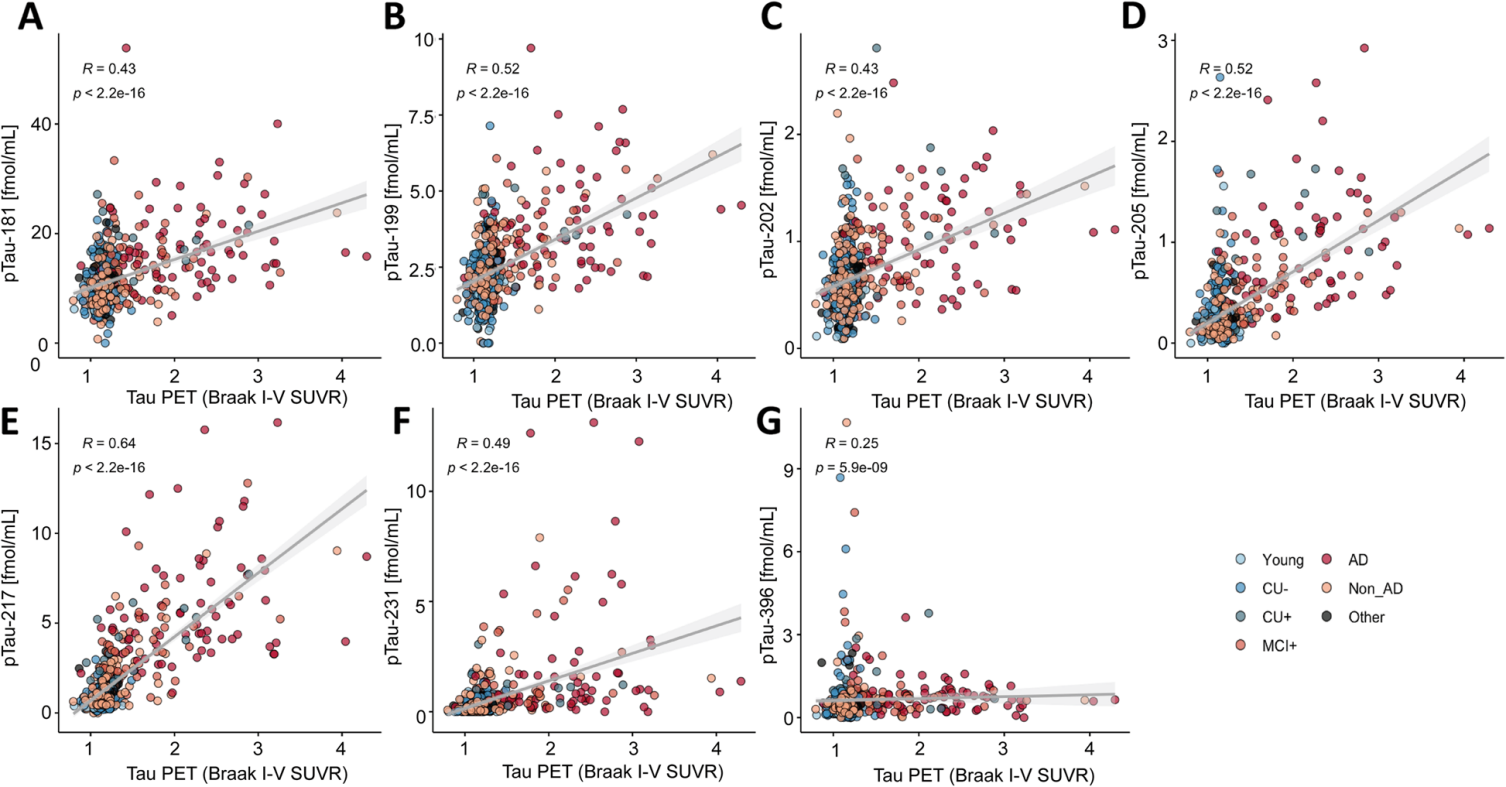
Correlation between the pTau biomarkers and tau PET (average SUVR of the regions corresponding to Braak stages I-IV) in the BioFINDER-2 cohort, for pTau-181 (**A**), pTau-199 (**B**), pTau-202 (**C**), pTau-205 (**D**), pTau-217 (**E**), pTau-231 (**F**) and pTau-396 (**G**) and tau pathology,. Abbreviations: Aβ, amyloid-β; AD, Alzheimer’s disease; CU-, Aβ-negative cognitively unimpaired; CU + , Aβ-positive cognitively unimpaired; frontotemporal dementia; MCI + , Aβ-positive mild cognitive impairment; Non-AD, Aβ-negative “AD” dementia or mild cognitive impairment patients; p, *P* value of the correlation test; PET, positron emission tomography; R, Spearman rank correlation coefficient; SUVR, standard uptake value ratio

A correlation analysis was also performed between the abundances of the different pTau epitopes and the average SUVR regions of Braak stage I-IV. Again, pT217, pT231 and pT205 showed the strongest correlation in both TRIAD (*ρ*_pTau-217_ = 0.71, *P*_pTau-217_ < 0.001; *ρ*_pTau-231_ = 0.70, *P*_pTau-231_ < 0.001; *ρ*_pTau-205_ = 0.71, *P*_pTau-205_ < 0.001; Fig. 5) and BioFINDER-2 (*ρ*_pTau-217_ = 0.64, *P*_pTau-217_ < 2.2*10^–16^; *ρ*_pTau-231_ = 0.49, *P*_pTau-231_ < 2.2*10^–16^; *ρ*_pTau-205_ = 0.52, *P*_pTau-205_ < 2.2*10^–16^; Fig. 6).

## Discussion

Recent studies using ultrasensitive immunoassays indicate that different pTau species (pT181, pT217 and pT231) in plasma samples perform very well to identify AD, and correlate with both Aβ and AD-type tau pathology as measured by PET [16, 20–22]. Studies showing very high (AUC 0.98) ability of plasma pT217 to discriminate Alzheimer’s disease from other neurodegenerative disorders [43], stronger correlation of pT217 than pT181 with tau PET [14], CSF and IP-MS data showing a higher magnitude of increase of plasma pT217 and stronger association with Aβ PET than pT181 [17], and CSF pT231 increasing at the early stage of the disease suggest that there may be disease associated differences across these pTau species and that measurement of specific phosphorylated species may be useful to track disease progression.

The PRM method described here is well suited to address this task as it enables multiple analytes to be measured in the same analytical run, thereby reducing analysis time, sample consumption, and importantly, also the effects of inter-run variation caused, e.g., by additional sample handling and freeze–thaw cycles. Furthermore, employing an antibody-independent sample preparation makes it possible to measure phosphorylation at potentially any site in tau.

The use of perchloric acid to purify tau by precipitating other sample proteins is well-established [44] and no studies have reported discrimination of specific processed or phosphorylated tau forms using this method. Absolute quantification of non-phosphorylated CSF tau using sample preparation based on perchloric acid precipitation has been previously reported [30, 45]. The high degree of correlation with Simoa data for pTau-181, pTau-217, and pTau-231 in our study is also an indication that there is no discrimination of any of the phospho-epitopes analyzed using the perchloric acid-based samples preparation. Perchloric acid-based sample preparation may thus be suitable to use in a reference method.

While IP-MS based methods can perform relative quantification, e.g., measuring the ratio of phosphorylated to non-phosphorylated peptide [28], absolute quantification presents a problem, as quantification is affected by the affinity of the used antibodies for the target protein. We found that the fold changes between Alzheimer’s disease and CU- patients for the phosphorylation ratios, pS199/S199, pS202/S202, pT205/T205, and pT217/T217, were all smaller compared with the fold changes for the concentrations of the corresponding peptides themselves, quantified using the isotope-labelled internal standards (Supplementary Figure S6). This finding is logical when considering the large number of papers showing that levels pTau and total tau in CSF both are increased in Alzheimer’s disease and correlate tightly.

We also found increased levels of tau phosphorylated at serine-396 in Alzheimer’s disease compared with controls, in line with neuropathological evidence [46]. A major component of neurofibrillary tangles in Alzheimer’s disease brains [47], Serine-396 is located at the extreme C-terminal part of the tau molecule. Previous studies have concluded that pTau-396 levels are not released into the CSF in quantities that are high enough to be reliably quantified, and so far only a single study published in 2002 reported increased levels of tau phosphorylated at 396 and/or 404 in CSF [9].

The PRM method also showed, in the discovery cohort, high diagnostic potential and differentiated between biomarker-positive Alzheimer’s disease patients from biomarker-negative controls with up to 99% accuracy. We found marked increase of all phosphorylated tau species, with the highest increases observed for pT217, pT231 and pT205. Further, CSF levels of pT181, pT217 and pT231 correlated tightly (*p* < 0.001) with Simoa measurements of the same pTau species using assays based on the N-terminal non-pTau antibody in the sandwich pair [16]. The correlations were also strong between the PRM measurements for the three peptides (*ρ* > 0.94). Together, these findings support the LC–MS approach as a valid strategy to target multiple pTau biomarkers concurrently in the same sample.

The finding that both plasma and CSF pTau is truncated into N-terminal to mid-domain fragments [17], suggests that not only which pTau variant is captured, but also which pTau fragment is measured (as governed by the assay setup), may control the performance of pTau immunoassays as well as IP-MS methods. With our antibody-free PRM method, since short tryptic peptides encompassing each phosphorylated site are detected, all tau fragments that contain a given phosphopeptide are included in quantification, in effect, making quantification independent on proteolytic processing of tau. While this has the advantage of making the measured entity well defined, it may not be advantageous in terms of biomarker performance in all cases. For example, the reported presence in CSF of short endogenous tau peptides containing pT181 that are not increased in Alzheimer’s disease [48], may decrease the performance of the PRM assay for this peptide, compared to pT181 immunoassays that require a longer tau fragment for detection.

## Conclusions

Our study confirms the tau phospho-epitopes pT231 and pT217 as markers of early Alzheimer’s disease pathology, and identifies pT205 as a marker that increases in importance later in AD. The presented PRM method is the first antibody-independent method to measure pTau species in CSF, making it a potential candidate for future development of a reference method.

## Supplementary Information

**Additional file 1: Supplementary Table S1. **Precursor and product ions used for quantification. **Supplementary Table S2.** Method reproducibility assessment. **Supplementary Table S3.** Performance of the pTau biomarkers in the biochemically characterized AD patients and controls. **Supplementary Table S4.** ROC curve analysis and fold-change to discriminate between amyloid positive and negative groups in TRIAD. **Supplementary Table S5.** ROC curve analysis and fold-change to discriminate between amyloid positive and negative groups in BioFINDER-2. **Supplementary Figure S1.** PRM chromatograms of the assayed tau phosphopeptides. **Supplementary Figure S2.** Scatter plots of phosphorylated peptide abundances in biochemically characterized AD patients and controls. **Supplementary Figure S3.** Correlations between the pTau PRM assay and Simoa immunoassay. **Supplementary Figure S4.** ROC curve analysis to discriminate between amyloid positive and negative groups in TRIAD. **Supplementary Figure S5.** ROC curve analysis to discriminate between amyloid positive and negative groups in BioFINDER-2. **Supplementary Figure S6.** Fold changes (AD versus CU-) for phospho-epitope concentrations compared with phosphorylation ratios. 

## Data Availability

Anonymized data will be shared by request from a qualified academic investigator for the sole purpose of replicating procedures and results presented in the article and as long as data transfer agrees with EU legislation on the general data protection regulation and decisions by the Ethical Review Board of the cohorts, which should be regulated in a material transfer agreement.

## Abbreviations

Aβ: Amyloid beta
AUC: Area under the curve
CDR: Clinical Dementia Rating
CU: Cognitively unimpaired
FC: Fold change
GLM: Generalized linear model
HSD: Tukey honestly significant difference
IP: Immunoprecipitation
LC: Liquid chromatography
MCI: Mild cognitive impairment;
MS: Mass spectrometry
PRM: Parallel reaction monitoring;
pTau: Phospho-tau
ROC: Receiver operating curve
SUVR: Standardized uptake values ratio
tTau: Total tau
