# Supplementary material for: Antibody-free measurement of cerebrospinal fluid tau phosphorylation across the Alzheimer’s disease continuum
Source: Mol Neurodegener. 2022 Dec 12;17:81. doi: 10.1186/s13024-022-00586-0 (PMC9743664; doi:10.1186/s13024-022-00586-0)

Supplementary data

Antibody-free measurement of cerebrospinal fluid tau phosphorylation across the Alzheimer's disease continuum

Contents

Supplementary Table S1. Precursor and product ions used for quantification

Supplementary Table S2. Method reproducibility assessment

Supplementary Table S3. Performance of the pTau biomarkers in the biochemically characterized AD patients and controls

Supplementary Table S4. ROC curve analysis and fold-change to discriminate between amyloid positive and negative groups in TRIAD

Supplementary Table S5. ROC curve analysis and fold-change to discriminate between amyloid positive and negative groups in BioFINDER-2

Supplementary Figure S1. PRM chromatograms of the assayed tau phosphopeptides

Supplementary Figure S2. Scatter plots of phosphorylated peptide abundances in biochemically characterized AD patients and controls

Supplementary Figure S3. Correlations between the pTau PRM assay and Simoa immunoassay

Supplementary Figure S4. ROC curve analysis to discriminate between amyloid positive and negative groups in TRIAD

Supplementary Figure S5. ROC curve analysis to discriminate between amyloid positive and negative groups in BioFINDER-2

Supplementary Figure S6. Fold changes (AD versus CU-) for phospho-epitope concentrations compared with phosphorylation ratios

Supplementary Table S1. Precursor and product ions used for quantification.

		Product m/z	Product Charge	Fragment Ion
**Phosphorylation Site**	Thr-181	341.2183	1	y3
**Peptide Sequence**	TPPAPK[pT]PPSSGEPPK	367.1976	1	b4
**Amino Acids**	175-190	490.5727	3	y14
**Precursor m/z**	556.6062	522.9236	3	y15
**Precursor Charge**	3	592.3453	1	b6
**HCD Collision Energy (%)**	26	602.3220	2	y12 -98
**Spike-in conc. (fmol/ml CSF)**	120	651.3105	2	y12
		675.3824	1	b7
		686.8290	2	y13
		686.3760	2	y14-98
		701.3464	1	y7
		735.3554	2	y14
		798.3992	1	y8
**Phosphorylation Site**	Ser-199	456.7303	2	y10
**Peptide Sequence**	SGYS[pS]PGSPGTPGSR	464.1776	1	b5 -98
**Amino Acids**	195-209	618.2518	1	b7 -98
**Precursor m/z**	737.3039	758.3791	1	y8
**Precursor Charge**	2	815.4006	1	y9
**HCD Collision Energy (%)**	35	912.4534	1	y10
**Spike-in conc. (fmol/ml CSF)**	8			
**Phosphorylation Site**	Ser-202	370.6879	2	y8 -98
**Peptide Sequence**	SGYSSPG[pS]PGTPGSR	399.1987	2	y9 -98
**Amino Acids**	195-209	447.725	2	y10 -98
**Precursor m/z**	737.3039	482.1882	1	b5
**Precursor Charge**	2	496.7135	2	y10
**HCD Collision Energy (%)**	35	740.3686	1	y8 -98
**Spike-in conc. (fmol/ml CSF)**	8	797.39	1	y9 -98
		894.4428	1	y10 -98
		992.4197	1	y10
**Phosphorylation Site**	Thr-205	376.1604	2	y7
**Peptide Sequence**	SGYSSPGSPG[pT]PGSR	416.2252	1	y4
**Amino Acids**	195-209	482.1882	1	b5
**Precursor m/z**	737.3039	496.7135	2	y10
**Precursor Charge**	2	751.3134	1	y7
**HCD Collision Energy (%)**	35	838.3455	1	y8
**Spike-in conc. (fmol/ml CSF)**	8	877.3686	1	b10
		895.3669	1	y9
		992.4197	1	y10
**Phosphorylation Site**	Thr-217	374.6731	2	y6
**Peptide Sequence**	TPSLP[pT]PPTR	399.2238	1	b4

**Amino Acids**	212-221	470.2722	1	y4
**Precursor m/z**	573.7814	523.2575	2	y9
**Precursor Charge**	2	748.3389	1	y6
**HCD Collision Energy (%)**	25	948.4550	1	y8
**Spike-in conc. (fmol/ml CSF)**	8			
**Phosphorylation Site**	Thr-231	368.7450	2	b7 -98
**Peptide Sequence**	KVAVVR[pT]PPKSPSSAK	417.7335	2	b7
**Amino Acids**	225-240	449.7533	2	y9
**Precursor m/z**	577.9887	489.2667	1	y5
**Precursor Charge**	3	618.8566	2	y12 -98
**HCD Collision Energy (%)**	32	668.3908	2	y13 -98
**Spike-in conc. (fmol/ml CSF)**	4	703.9093	2	y14-98
		717.3792	2	y13
		736.4828	1	b7 -98
		752.8978	2	y14
		753.4436	2	y15 -98
		801.4465	1	y8
		802.4320	2	y15
		898.4993	1	y9
**Phosphorylation Site**	Thr-396	460.2514	1	y4
**Peptide Sequence**	TDHGAEIVYK[pS]PVVSGDTSPR	482.1994	1	b5
**Amino Acids**	386-406	507.7644	2	y10
**Precursor m/z**	765.6933	542.2751	2	y11-98
**Precursor Charge**	3	611.2420	1	b6
**HCD Collision Energy (%)**	30	632.2998	1	y6
**Spike-in conc. (fmol/ml CSF)**	4	693.6684	3	y19
		818.4003	1	y8
		823.3945	1	b8
		1014.5214	1	y10
		1083.5429	1	y11-98

Supplementary Table S2. Method reproducibility assessment. Aliquots of a CSF pool was prepared separately and analyzed at 8 occasions over two days.

Analyte	Mean conc. [fmol/ml]	CV [%]
pTau-181	17.75	7.35
pTau-199	2.68	3.80
pTau-202	1.04	3.10
pTau-205	1.32	12.78
pTau-217	1.39	19.01
pTau-231	1.79	10.50
pTau-396	0.41	14.89

Supplementary Table S3. Performance of the pTau biomarkers in biochemically characterized AD patients and controls. 'C' = Controls; AUC = Area under the ROC curve.

Analyte	Mean Conc. AD [fmol/ml]	Mean Conc. C [fmol/ml]	mean fold change	AUC	Cohen's d
pTau-181	26.25	13.52	1.942	1.000	2.414
pTau-199	04.01	2.22	1.807	0.953	2.395
pTau-202	01.70	0.85	2.000	0.956	2.145
pTau-205	2.19	0.62	3.537	0.994	2.508
pTau-217	4.01	0.52	7.745	1.000	3.192
pTau-231	4.01	0.60	6.700	1.000	2.319
pTau-396	0.64	0.41	1.536	0.831	1.303

Abbreviations: AUC, area under the ROC curve.

Supplementary Table S4. ROC curve analysis to discriminate groups in the TRIAD cohort.

	**Aβ-PET positive *vs* CU-**		**AD *vs* CU-**		**MCI+ *vs* CU-**		**CU+ *vs* CU-**	
	**AUC (95% CI)**	**Fold-change**	**AUC (95% CI)**	**Fold-change**	**AUC (95% CI)**	**Fold-change**	**AUC (95% CI)**	**Fold-change**
pTau-181	75.41 (66.68-84.13)	1.41	79.63 (68.28-90.98)	1.52	80.99 (69.57-92.42)	1.56	68.23 (55.58-80.89)	1.23
pTau-199	77.75 (69.4-86.1)	1.44	88.17 (77.31-99.02)	1.76	81.09 (70.11-92.08)	1.44	68.09 (54.87-81.31)	1.21
pTau-202	73.78 (64.7-82.86)	1.39	83.85 (73.51-94.19)	1.63	74.17 (61.29-87.05)	1.43	66.52 (53.41-79.63)	1.18
pTau-205	85.07 (77.79-92.34)	3.35	99.07 (97.21-100)	5.34	90.25 (80.23-100)	3.96	71.58 (58.29-84.87)	1.51
pTau-217	92.8 (87.78-97.81)	4.26	99.71 (99.06-100)	6.33	98.93 (97.36-100)	4.73	83.26 (72.15-94.37)	2.39
pTau-231	93.61 (89.61-97.6)	5.72	98.73 (96.99-100)	7.64	98.54 (96.63-100)	6.44	86.25 (78.15-94.35)	3.80
pTau-396	69.14 (59.58-78.69)	1.37	63.99 (48.72-79.26)	1.28	79.04 (66.62-91.47)	1.52	65.46 (52.09-78.82)	1.33

Supplementary Table S5. ROC curve analysis to discriminate groups in the BioFINDER-2 cohort.

	Aβ+* *vs* CU-		AD *vs* CU-		MCI+ *vs* CU-		CU+ *vs* CU-	
	**AUC (95% CI)**	**Fold-change**	**AUC (95% CI)**	**Fold-change**	**AUC (95% CI)**	**Fold-change**	**AUC (95% CI)**	**Fold-change**
pTau-181	77.87 (73.29-82.45)	1.58	85.37 (79.68-91.05)	1.87	76.31 (68.7-83.90)	1.54	76.43 (69-75-83.12)	1.41
pTau-199	76.18 (71.50-80.87)	1.58	87.12 (82.27-91.96)	1.99	76.03 (68.79-83.27)	1.51	66.54 (59.04-74.04)	1.31
pTau-202	70.63 (65.52-75.74)	1.52	81.38 (75.38-87.38)	1.86	69.05 (61.21-76.90)	1.41	62.86 (54.92-70.80)	1.35
pTau-205	81.99 (77.77-86.20)	2.50	93.51 (90.16-96.85)	3.92	82.55 (76.45-88.66)	2.11	71.11 (63.86-78.36)	1.70
pTau-217	94.50 (92.40-96.61)	5.45	98.12 (95.70-100.00)	8.83	94.79 (91.18-98.41)	4.86	91.05 (86.82-95.27)	3.29
pTau-231	84.44 (80.57-88.31)	6.40	91.89 (88.37-95.41)	10.65	85.45 (80.36-90.54)	5.62	78.69 (72.27-85.12)	3.65
pTau-396	69.97 (64.78-75.16)	1.30	72.02 (64.63-79.42)	1.33	74.31 (66.52-82.10)	1.59	69.30 (61.95-76.65)	1.21

* CSF Ab42/40 ratio

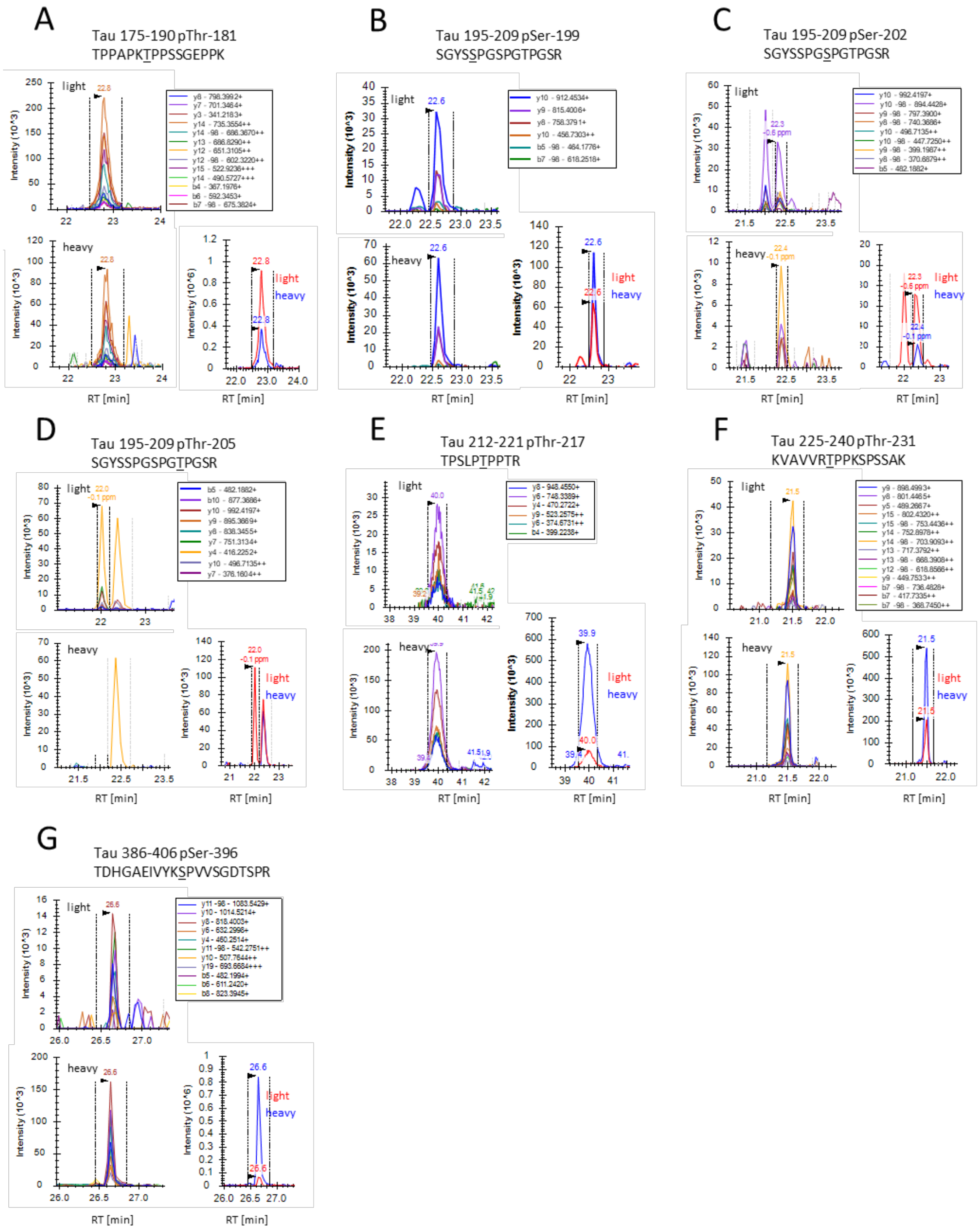

Supplementary Figure S1. Representative PRM chromatograms of the assayed tau phosphopeptides, Tau 175-190 p181 (A), Tau 195-209 p199 (B), Tau 195-209 p202 (C), Tau 195-209 p205 (D), Tau 212-224 p217 (E), Tau 225-240 p231 (F), Tau 386-406 p396 (G). For each phosphopeptide, the chromatographic traces of the included b- and y- fragment ions used for quantification are indicated in the two left panels, for the light (top) and heavy (bottom) form. The summed up peak areas of these traces for the light and heavy forms, used to calculate the light-to-heavy ratio, is shown in the bottom right panel.

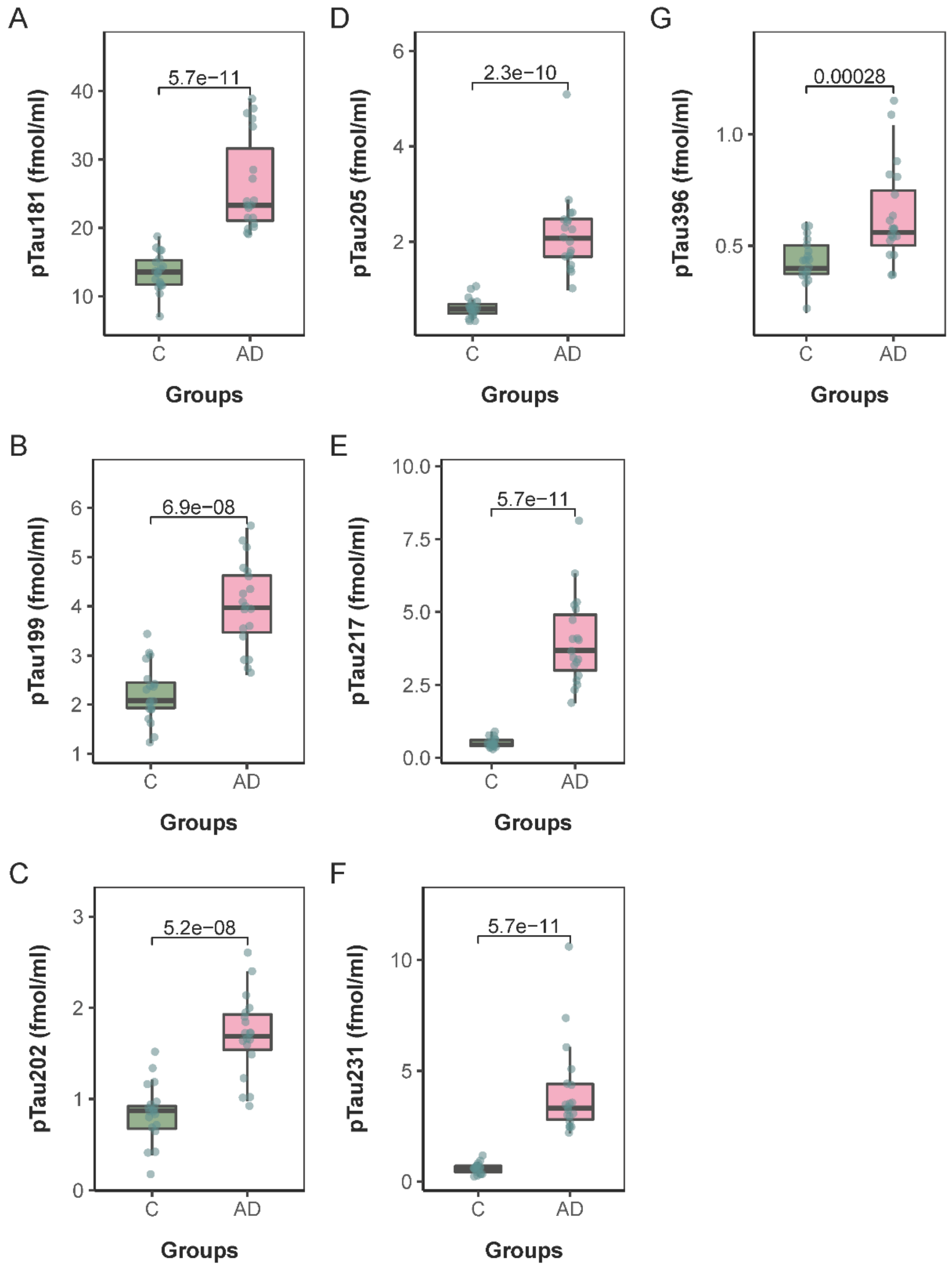

Supplementary Figure S2. Scatter plots of phosphorylated peptide abundances in biochemically characterized AD patients and controls. The phosphorylated epitopes measured were Thr-181 (A), Ser-199 (B), Ser-202 (C), Thr-205 (D), Thr-217 (E), Thr-231 (F) and Thr-396 (G). Concentrations are given as femtomoles of the respective measured tryptic phospopeptide per mL CSF. The boxplots depict the median (horizontal bar), interquartile range (IQR, hinges) and 1.5 x IQR (whiskers). Abbreviations: AD, Alzheimer’s disease; C, control.

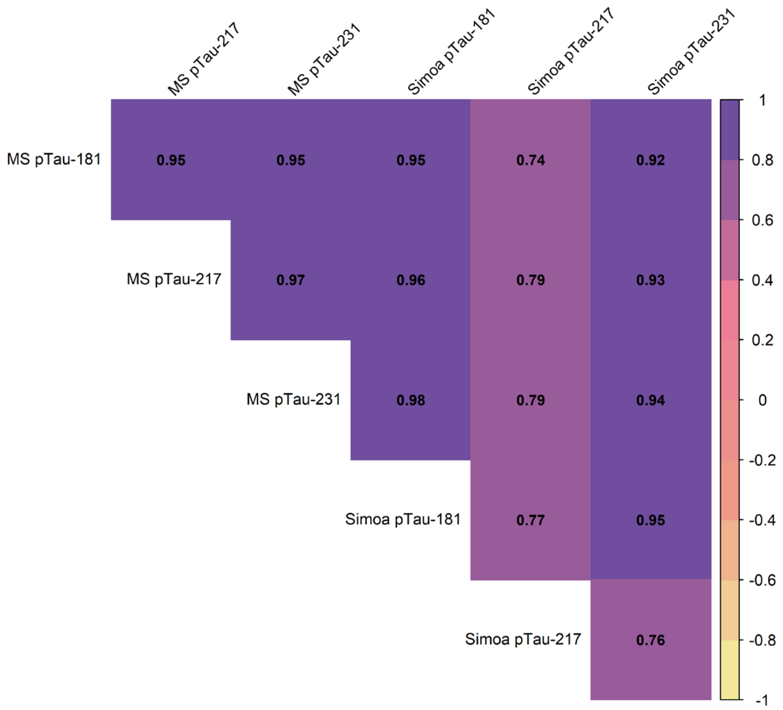

Supplementary Figure S3. Correlations (Spearman) between the pTau PRM assay and Simoa immunoassay for pTau-181, pTau-217 and pTau-231.

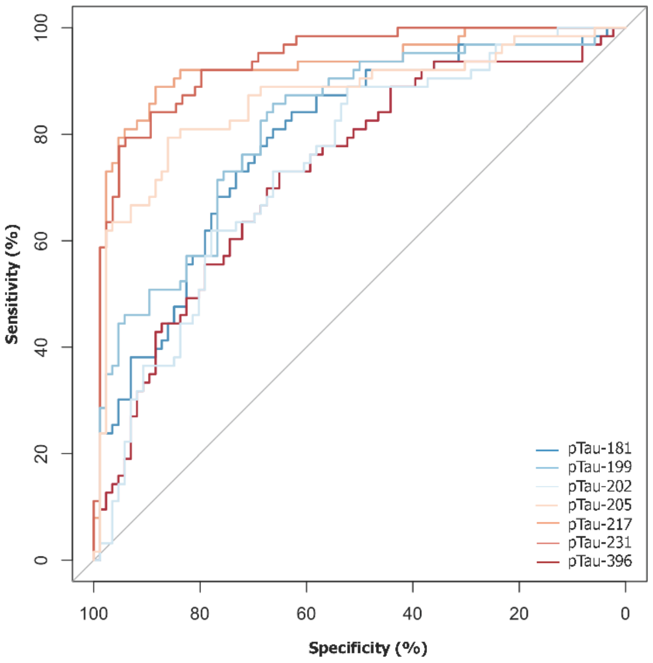

Supplementary Figure S4. Discriminative accuracy of pTau biomarkers in the TRIAD cohort. ROC curve analysis of the performance of pTau-181, pTau-119, pTau-199, pTau-202, pTau-205, pTau217, pTau-231, and pTau-396 for distinguishing between Aβ positive and negative individuals (as indicated by Aβ PET).

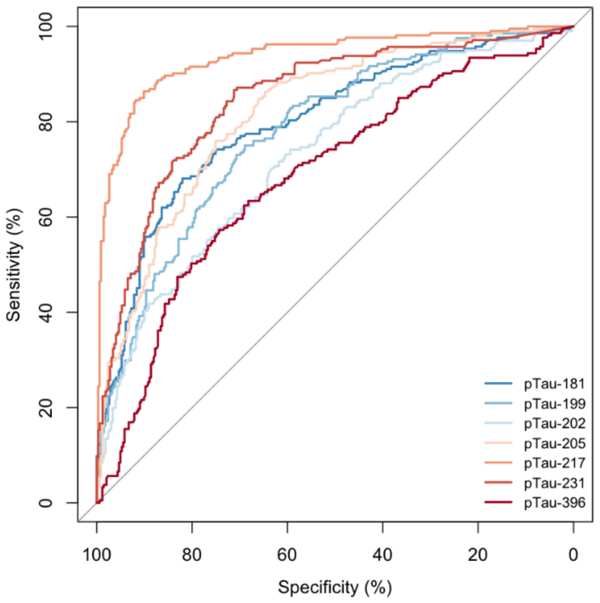

Supplementary Figure S5. Discriminative accuracy of pTau biomarkers in the BiofFINDER cohort. ROC curve analysis of the performance of pTau-181, pTau-119, pTau-199, pTau-202, pTau-205, pTau217, pTau-231, and pTau-396 for distinguishing between Aβ positive and negative individuals (as indicated by Aβ PET).

Supplementary Figure S6. Fold changes in AD versus CU- cases for the phospho-epitopes pS199, pS202, pT205 and pT217, and for the phosphorylation ratios pS199/S199, pS202/S202, pT205/T205 and pT217/T217, measured relative to the corresponding non-phosphorylated peptides.

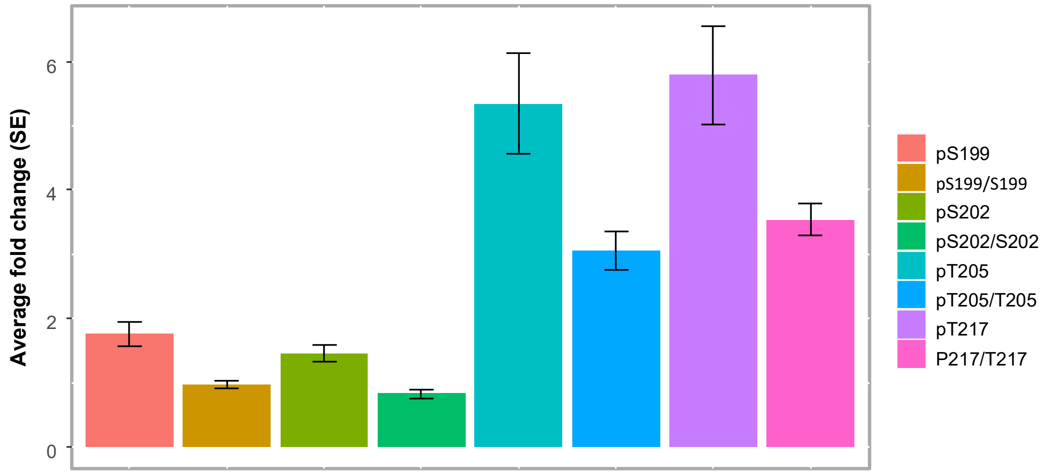

Supplement: Supplementary file 1 — Additional file 1: Supplementary Table S1. Precursor and product ions used for quantification. Supplementary Table S2. Method reproducibility assessment. Supplementary Table S3. Performance of the pTau biomarkers in the biochemically characterized AD patients and controls. Supplementary Table S4. ROC curve analysis and fold-change to discriminate between amyloid positive and negative groups in TRIAD. Supplementary Table S5. ROC curve analysis and fold-change to discriminate between amyloid positive and negative groups in BioFINDER-2. Supplementary Figure S1. PRM chromatograms of the assayed tau phosphopeptides. Supplementary Figure S2. Scatter plots of phosphorylated peptide abundances in biochemically characterized AD patients and controls. Supplementary Figure S3. Correlations between the pTau PRM assay and Simoa immunoassay. Supplementary Figure S4. ROC curve analysis to discriminate between amyloid positive and negative groups in TRIAD. Supplementary Figure S5. ROC curve analysis to discriminate between amyloid positive and negative groups in BioFINDER-2. Supplementary Figure S6. Fold changes (AD versus CU-) for phospho-epitope concentrations compared with phosphorylation ratios. [file 13024_2022_586_MOESM1_ESM.pdf]
